# Use of Digital Technology among Adolescents Attending Schools in Bissau, Guinea-Bissau

**DOI:** 10.3390/ijerph17238937

**Published:** 2020-12-01

**Authors:** Geir Gunnlaugsson, Thomas Andrew Whitehead, Fatou N’dure Baboudóttir, Aladje Baldé, Zeca Jandi, Hamadou Boiro, Jónína Einarsdóttir

**Affiliations:** 1 Faculty of Sociology, Anthropology and Folkloristics, University of Iceland, IS-102 Reykjavík, Iceland; taw5@hi.is (T.A.W.); fnb1@hi.is (F.N.B.); hboiro@gmail.com (H.B.); je@hi.is (J.E.); 2 University Campus, Jean Piaget University of Guinea-Bissau, Bissau 5100, Guinea-Bissau; aladje@gmail.com; 3 Instituto Nacional de Estudos e Pesquisa (INEP), Avenida dos Combatentes da Liberdade da Pátria, Complexo Escolar 14 de Novembro, Bissau C.P. 112, Guinea-Bissau; jandizeca@gmail.com

**Keywords:** survey, Africa, sub-Sahara, least developed countries, socioeconomic factors, school-age population, internet access, computer hardware, mobile phone

## Abstract

Digital technology plays an important role in achieving many of the Sustainable Development Goals. However, access is uneven, with 80% of those in high-income countries being online compared to 20% of those in the 47 least developed countries. This study aimed to describe and analyse adolescents’ access to and usage of digital technology in Guinea-Bissau and its implications. In June 2017, a survey with a locally adapted Planet Youth questionnaire was implemented in the capital, Bissau, whereby classes in 16 secondary schools were surveyed on a variety of issues. In total, 2039 randomly selected students participated; the survey included ten questions specifically on the access to and use of digital technology. Half of the respondents had access to desktop/laptops, and one-third used mobile internet daily; about two-thirds had an experience of social media. Explanatory variables included educational institution, parental education, economic situation, and gender. Furthermore, students’ experience of social media was significantly linked to bullying, anxiety, depression, smoking and alcohol consumption. Many adolescents in Bissau have no experience of using digital technology, including for schoolwork. Access improvements are necessary so that young Bissau-Guineans are not to be left behind in developing their capabilities and can benefit from proficiency in the use of digital technologies. At the same time, potential harmful usage of the media requires the implementation of preventive measures.

## 1. Introduction

With the launch of the Sustainable Development Goals 2016–2030 (SDGs), world leaders pledged to leave no one behind and called for integrated action across sectors to tackle complex development challenges [1]. Since its adoption, adolescents have increasingly been in focus for global health policy to support the ‘SDG generation’ to realise their rights to health, wellbeing, education and full participation in society, and thereby enable them to attain their full potential as adults [2,3,4,5]. 

The focus on youth is important for several reasons. First and foremost, this generation will be the world leaders of the future, making important political and economic decisions which impact global development. Secondly, the number of people aged 10–24 is the largest in history, standing at over 1.8 billion as of 2016 [6]. Thirdly, adolescents are “biologically, emotionally and developmentally primed for engagement beyond their families and the media, in particular social media, offers that opportunity” [7,8]. Digital technology plays an important role in achieving the SDGs, not only through Goal 9 (Industry, innovation, and infrastructure), but as a driver for the fulfilment of all the goals. This is important for young people who currently are the most connected demographic globally, with 71% of 15–24-year-olds being online, compared to 48% of the total population, and those under the age of 18 making up around one-third of all internet users around the world [9]. However, global access is uneven, causing a digital divide [9,10,11]; African youth are the most disconnected in the world, with over 60% not being online, compared to just 4% of those in Europe [9]. Nonetheless, the proportion of Africans with internet access is growing rapidly, from 0.02% in 1998 to 10% in 2010 [12], and 28% in 2019 [13]. Inequality remains a challenge [14] and must be addressed for the achievement of the SDGs; in 2019, about 34% of African men had access to the internet compared to 23% of females [13].

### 1.1. Uses of Digital Technology—Pros and Cons

Digital technology refers to the use of mobile phone devices, computers/laptops with and without internet capabilities and social media that is accessed on those devices. There is an array of literature outlining what it is that youth across the world are using these modern devices to do, as well as the positive and negative consequences of these uses. 

Commonly mentioned positive usages of digital technology among adolescents include communicating with friends and family [15,16,17], identity building [10,18,19], educational purposes [9,20], finding information [21,22], entertainment [16,17], and job hunting [15,23]. Lesser mentioned positives include spreading awareness of world issues and increased activism [24,25] and promoting tourism in their countries [26]. Negative usages include cyberbullying [9,10,23], worries over a “bedroom culture” being formed with adolescents staying in their rooms roaming the internet [9], and increasing fears over the morale of adolescents with specific reference to body-image [7,9]. Its usage also facilitates the promotion of unhealthy lifestyle choices and unwanted sexual content [9,23], and gambling [27].

In sub-Saharan Africa (SSA), the literature on how digital technologies are accessed overwhelmingly highlight mobile devices being used and owned more commonly than computers or laptops [12,13,26]. Although computers are important tools for internet usage, mobile phones offer access to a more varied audience. One common trend that influences the preference of phones over computers is the concept of phone sharing, whereby many individuals (friends or family) share usage of one handset, often switching out SIM cards for their own [17,19,28]. Furthermore, the number of mobile money service subscribers is increasing rapidly across SSA [29].

### 1.2. Determinants and Barriers to Usage

Determinants for access to different forms of digital technologies include gender, education levels of adolescents and rural/urban habitation. These, among other reasons, create what has been dubbed “digital divides” that mirror prevailing economic gaps [9]. Compared to African youth where over 60% are not online [9], 90% of adolescents in the USA used at least one form of social media [30] and 92% of adolescents aged 14–16 years used it for at least 30 min each day in Iceland [31]. Furthermore, globally there is a gender gap of 17% with more males using the internet than females, as of 2019, and has increased by 6% compared to 2013 [13]. Although most countries in Africa are reporting large gender gaps, South Africa appears to be the one country in the region that is closing the gender gap [19,23]. Furthermore, a higher proportion of people living in urban areas of African countries reported usage of digital technologies, compared with those living in rural areas [16,17,32]. 

Concerning intangible barriers that are blocking access to technology, one main obstacle to digital technology adoption is cost. High prices of technology compared to average incomes within SSA are well known [11,15,28,33]. Besides, poor infrastructure across the continent, especially in rural SSA, hinders accessibility. Finally, other barriers to usage include a lack of digital skills on the continent and large proportions of the population speaking minority languages; the majority of the content available online is in major languages that are not always understood in some parts of the world [9].

### 1.3. Background to Study

Guinea-Bissau is a low-income country located in west Africa, ranked as 178 out of 189 in the Human Development Index 2019 [34], 173 out of 182 countries on the Kids Rights Index 2020 [35], and belongs to a group of fragile states [36,37]. It became independent in 1974 after 11 years of the liberation war against the Portuguese. The country suffered from a military-political conflict in 1998–1999 and has since been characterised by political instability [36,38,39,40,41,42] with links to narco-trafficking [43]. The Bissau-Guinean population is estimated to be around 1.9 million and is rapidly growing, with the capital Bissau as the most populous area where about a quarter of them live [44]. About 40% are aged 0–14 years and one-third of young people aged 10–24 years [45]. Based on data from the most recent census (2009), adolescents aged 14–19 years comprised about a quarter of the Bissau population. The country is characterised by ethnic and cultural diversity. About 45% of the population adheres to Islam (mostly Fula, Mandinga and Beafada) [44]. Other ethnic groups (Balanta, Manjaco, Mancanha and Papel people) are the largest, in addition to smaller groups such as Bijagós, Felupe and Mansoanca) who adhere to the Christian religion (22%; Catholics and diverse Protestant/Evangelic churches) and various local religious practices. The official language, Portuguese, is spoken by 11–14%, most of whom belong to the educated elite in the capital [46]. Kriol, a Portuguese-based Creole language, de facto lingua franca, is spoken as a first language by about 15% [46], and today it is estimated that about 90% of the population speak Kriol [47]. Although only a small proportion of children speaks Portuguese, it is the language of teaching at all levels of education [48]. Irrespective of school form, either public or private, the curriculum is the same in line with guidelines from the Ministry of Education.

Almost four out of ten primary school-age children, irrespective of gender, are out-of-school [49], and fewer than one-third of children start school at the correct age [50]. Only 14% of children who enrol in grade 1 complete grade 12 in secondary schools [51]. Based on information from the Ministry of Education and Higher Education for the academic year 2014–2015 (most recent available), in total there were 31,387 students registered in the 7th to 10th grade in public and private schools in Bissau, of whom 51.6% were boys [52].

### 1.4. Aims of the Study

In the context of schools in a low-income country, here Guinea-Bissau, the aims are to describe, explore and analyse socioeconomic determinants for adolescents´ access to and usage of digital technology, and its implications. We hypothesise that in this privileged group of adolescents’, access will be uneven, shaped by socioeconomic conditions, while the impact of its use is shared with adolescents elsewhere.

## 2. Materials and Methods 

### 2.1. Implementation of the Survey

The data collection followed a distinct and well-established methodology of the Youth in Europe collaboration, called Planet Youth since 2015, and led by the Icelandic Centre for Social Research and Analysis (ICSRA) [53,54,55,56]. All the survey questions had earlier been developed by an interdisciplinary team of professionals and used for more than a decade by the collaborating partners to conduct surveys among adolescents aged 14–16 years across Europe and elsewhere. Encouraged by this experience, the survey questions were translated into Portuguese, pilot-tested and adapted to the context in Guinea-Bissau, e.g., in terms of the language used, and shortened to facilitate completion within the assigned 90 min (two classroom sessions). The final questionnaire included 312 questions divided into 77 main themes, including the use of digital technology. Questions on their socioeconomic background were no. 1–20 and 38, on digital media no. 29 and 30, mental health no. 45, smoking no. 55, and alcohol no. 61. Although the study was the first of its kind in the setting, after the initial introduction at the beginning of the class, the students rapidly fell into a rhythm and delivered filled-in questionnaires within 60–90 min. When it was enquired in class how it was going, the students said it was easy for them to answer the questions and expressed satisfaction to have been given the opportunity to participate. 

The target age-group for the study was adolescents aged 15–16 years, randomly selected by a methodology applied within the Youth in Europe network [54]. Firstly, two of the authors (GG, ZJ) identified 17 educational institutions that offered education for this age-group in the capital Bissau. Contact was made with the headmasters who were invited with two teachers for a preparatory meeting to explain and discuss the study and its background. In March 2017, GG and ZJ visited the 17 schools to present and discuss once again the study on-site with school headmasters and teachers. Class lists were not accessible in a computerised form except in a few cases, therefore the authors compiled an Excel file with the support of collaborators with information on all classes in the 17 schools that included at least one-third of students within the target age-group. In total, the file included 116 classes with 4470 students enrolled in grades 7–10, or about 20% of the approximately 24000 students who were projected to have been enrolled at the time. Thereafter, a random number was electronically assigned to each class in the Excel file on which their selection for participation was based [54].

The study was implemented in June 2017; after randomisation of classes, one private school chose not to participate due to ongoing final examinations. In total, 12 public schools and four private ones participated in the study, two thereof had links to Catholicism, one to Islam, and one without a religious affiliation. In total, 2110 randomly selected students took part in the survey, or 47.2% of the 4470 students who were included in the specially compiled class list. 

All completed questionnaires were sent to Iceland for electronic data entry scanning by ICSRA at Reykjavík University. In total, 2039 questionnaires were successfully digitised and made up the total number of adolescents who participated in the survey. Unsuccessful scanning was, for example, due to fine-grained, red dust on the questionnaires from the field setting. For each question asked, there was a minority of students who did not answer and were excluded in the analysis for that particular question; no question had an answer from all the 2039 students, except school name.

### 2.2. Statistics

After digitisation, data were imported into the IBM SPSS Statistics 21.0 (Armonk, New York, NY, USA) by ICSRA staff and codified with numerical values. The data files were then imported into JMP 14.3 (© 2020 SAS Institute Inc. Cary, NC, USA) for data analysis; “don´t know/doesn’t apply” answer, and missing values were excluded from the analysis. Firstly, we conducted descriptive statistics on all study variables. Thereafter, each of the variables was recoded to a nominal variable with a value of either 1 or 0 for bivariate analysis to identify significant explanatory variables for each dependent variable. The likelihood ratio Chi-square test was used (*p* < 0.05) and odds ratios (OR) calculated with 95% confidence intervals (CI) was used to evaluate statistical significance. For each of the dependent variables, potential explanatory variables were then introduced into a multivariate nominal logistic regression model. Non-significant variables were gradually removed from the model, and an R-squared value (*R*^2^) was calculated for the final model.

### 2.3. Ethics

The study was approved on 6 June 2017, by the Minister of Education in Guinea-Bissau (No/Ref 250/MEES/GM/2017), in line with national requirements. Furthermore, school headmasters approved the implementation in their respective schools, as did the teachers. Most of the participants were aged 14–17 years; thus, they were children as defined by the Convention on the Rights of the Child (CRC) [57]. In line with the CRC, children have the right to express their opinion on their situation and participate as found appropriate for their age. In the setting, demand for prior parental consent might result in biased data and low response rate [58,59]. Furthermore, scholars have called for socio-culturally responsive ethics reviews [60,61]. Consequently, prior parental consent was not requested.

Before the implementation of the survey in the classroom, students were informed that their participation was voluntary, the survey questions were not an examination, and they were allowed to skip answers to any or all of the questions. To ensure anonymity, no student revealed his/her identity on the questionnaire, and upon completion, each student put his/her questionnaire in an envelope and sealed it before handing it over to the teacher or the authors (GG/ZJ, HB, JE). 

## 3. Results

Table 1 details the demographic background of the respondents (Table S1 with survey questions). There was a roughly even gender split, with slightly more female participants (52%) than male. The majority of participants were aged 15–17 years and had a mother tongue that was not Portuguese.

In the questionnaire, there were 10 questions regarding the usage of digital technology. On average, 1498 (median 1464, range 1293–1705) out of 2039 (74%) participants reported on each of the ten questions on digital experience. Across the 10 questions, on average, 45% (median 46, range 21–69) of the respondents reported that they had never used the cited technology.

### 3.1. Access to Digital Technology

Table 2 shows the split between the usage of laptops, mobile phones with internet and desktop computers, by gender (Table S2, by public and private school). Respondents had the most experience in using mobile technology with an internet connection (79%), while the use of a desktop computer (31%) was the least frequent experience. About 10% reported that they had no experience of using any of the cited digital technologies.

### 3.2. Usage of Desktop Computer and/or Laptop

Fifty-nine percent of the participants reported that they had never used a laptop, and 69% had never used a desktop computer. In total, 75% of the participants responded to one or both questions regarding their use of a laptop or desktop computer; 51% had access to at least desktop computer or a laptop, thereof 28% had access to both technologies (Table 2 and Table S2). Results of the bivariate analysis are shown in Table 3 for nine socioeconomic explanatory variables for the use of laptops and/or desktop computers.

The nine socioeconomic variables, as spelt out in Table 3, were subsequently introduced into a multivariate nominal logistic regression model to identify explanatory variables for the dependent variable ‘use of computer and/or laptop’, with non-significant variables at 0.05 significance level gradually removed (*R*^2^ = 0.0769, *p* < 0.0001). The most significant variables for adolescents´ access to a desktop and/or a laptop were coming from a family that could afford a car (OR 1.95, 95% CI 1.51–2.53; *p* < 0.0001), attending a private school (OR 1.89, 95% CI 1.44–2.49; *p* < 0.0001), coming from a two-headed family (OR 1.76, 95% CI 1.36–2.27; *p* < 0.0001) and having at least one parent who had started or completed university education or technical training (OR 1.46, 95% CI 1.10–1.94; *p* = 0.0087).

### 3.3. Usage of Mobile Phone with Internet Capability

Use of mobile phones with an internet connection was reported by 79% of the participants (Table 2 and Table S2). Statistically significant explanatory variables in the bivariate analysis for access to this technology were gender (boy), attending private school, having at least one parent who had started or completed university education or technical training, reporting that Portuguese was spoken in the home, and belonging to a family that could afford to buy a car (Table 3). In a multivariate nominal logistic regression model introducing the nine explanatory variables in Table 3, the final statistically significant model (*R*^2^ = 0.0294, *p* < 0.0001) for the usage of a mobile phone with internet capabilities included gender (boy) (OR 1.70, 95% CI 1.31–2.21; *p* < 0.0001), attending a private school (OR 1.83, 95% CI 1.39–2.42; *p* < 0.0001), and coming from a family that could afford a car (OR 1.34, 95% CI 1.02–1.75; *p* = 0.0348).

### 3.4. Usage of Common Applications of Digital Media

Participants were asked several questions about their usage of common applications of digital media in the last 12 months (Table 4 by gender and Table S3 by public and private school). In total, 46 (2%) did not respond to any of the questions on the use of digital communication across the seven different forms of usage. Of those who responded at least once, 33% did not report any experience at all in one or more of the tools of digital technology (Table 4), that is 35% of the boys and 31% of the girls.

Usage of digital media in the last 12 months was grouped into three different categories: for studies; any use of social media; and entertainment, games and following the news (Table 5).

The most statistically significant explanatory variables in the bivariate analysis for any usage of the internet for studies in the last 12 months were gender (boys), living in a two-headed household, and coming from a family that could afford a car. In a multivariate nominal logistic regression model, introducing the explanatory variables in Table 5, the final statistically significant model for using the internet for studies (*R*^2^ = 0.0106, *p* < 0.0001) included gender (boy) (OR 1.47, 95% CI 1.19–1.82; *p* = 0.0003), and coming from a family that could not afford a car (OR 1.36, 95% CI 1.10–1.68; *p* = 0.0039).

For the use of social media in the last 12 months, the most significant explanatory variables were gender (boy), being born in Bissau, attendance of a private school, coming from a family with one working caretaker, living in a home where Portuguese was spoken, and whose family could afford a car (Table 5). In a multivariate nominal logistic regression introducing the explanatory variables in Table 5, the final statistically significant model for the use of social media (*p* < 0.0001, *R*^2^ = 0.0376) included attending private school (OR 1.71, 95% CI 1.31–2.24; *p* = 0.0013), gender (boy, OR 1.50, 95% CI 1.17–1.93; *p* = 0.0013), coming from a family that could afford a car (OR 1.49, 95% CI 1.15–1.93; *p* = 0.0023), and being born in Bissau (OR 1.35, 95% CI 1.03–1.76; *p* = 0.0272).

Use of digital media for entertainment, playing games and/or following the news in the last 12 months was statistically significant for gender (boy), being born in Bissau, attendance of private school, education of parents (university studies or technical training), and belonging to a family where Portuguese was spoken at home and that could afford to buy a car. In a multivariate nominal logistic regression, introducing the explanatory variables in Table 5, the final statistically significant model for the use of digital media for entertainment, playing games and/or following the news (*p* < 0.0001, *R*^2^ = 0.0499) included gender (boy) (OR 2.53, 95% CI 1.98–3.24; *p* < 0.0001), attending a private school (OR 1.79, 95% CI 1.40–2.30; *p* < 0.0001), and being born in Bissau (OR 1.43, 95% CI 1.10–1.85; *p* = 0.0060).

When the usage of digital media for studies, social media and/or entertainment, games and following the news was combined, multivariate nominal logistic regression identified four highly significant explanatory variables for digital media usage among adolescents in Bissau (*R*^2^ = 0.0409, *p* < 0.0001), which were gender (boy, OR 2.18, 95% CI 1.64–2.88; *p* < 0.0001), attending a private school (OR 1.57, 95% CI 1.17–2.88; *p* = 0.0027), being born in Bissau (OR 1.45, 95% CI 1.09–1.94; *p* = 0.0107), and coming from a family that could afford to buy a car (OR 1.33, 95% CI 1.00–1.77; *p* = 0.0478).

### 3.5. Impact of Experience of Social Media

Five distinct survey questions were selected to analyse the potential association of experience of usage of social media and health and wellbeing of Bissau-Guinean adolescents, which were on bullying behaviour, depression, anxiety, smoking cigarettes and drinking alcohol, in addition to language proficiency. In the analysis, the experience of communicating in the last 12 months on digital media with friends, family, and people the respondents would like to know was included in addition to other explanatory variables (Table 5).

*Bullying.* In the survey, the following question was asked: How often (i.e., never, once, twice, 3–4 times, and five times or more) during the last 12 months, have you been part of a group that teased another? In total, 11% out of 1454 participants reported experience of social media and had been part of such a group. Those who had experienced the use of social media were 2.51 times (95% CI 1.59–3.97) more likely to report participation in bullying behaviour compared to those with no experience of social media. They were also more likely to attend a private school (OR 1.39, 95% CI 1.02–1.90; *p* = 0.0348) and come from a family where at least one parent had university or technical training (OR 1.72, 95% CI 1.21–2.50; *p* = 0.0020). There were no gender differences in the experience of bullying (*p* = 0.6730). In a multivariate nominal logistic regression model (*R*^2^ = 0.0244, *p* < 0.0001), the experience of social media was the most important explanatory variable for bullying (OR 2.4, 95% CI 1.49–3.90; *p* = 0.0003), in addition to having at least one parent who had university or technical training (OR 1.60, 95% CI 1.09–2.34; *p* = 0.0156).

*Depression*. In the survey, the following question was asked: How often (i.e., almost never, seldom, sometimes, and often) have you felt sad or blue in the past week? In total, 8% of 1440 participants reported having “often” felt sad or blue in the past week. Those who had the experience of social media were 1.62 times more likely (95% CI 1.01–2.63) to have often felt sad or blue in the past week compared to those who had no such experience. In a multivariate nominal logistic regression model, two variables were statistically significant for symptoms of depression (*R*^2^ = 0.0145, *p* = 0.0037). Girls were 1.73 times more likely (95% CI 1.16–2.58; *p* = 0.0074) compared to boys to have felt depressed in the past week and those with experience of social media were 1.69 times more likely (95% CI 1.04–2.74; *p* = 0.0353) to have felt depressed compared to those with no experience.

*Anxiety*. In the survey, the following question was asked: How often (i.e., almost never, seldom, sometimes, and often) have you felt tense the past week? In total, 8% out of 1409 participants reported having “often” felt tense the past week. Those who had an experience of social media were 1.80 times more likely (95% CI 1.10–2.93) to report to have often felt tense in the past week compared to those who had no experience, and multivariate nominal logistic regression analysis did not identify other explanatory variables.

*Smoking*. In the survey, the following question was asked: How often have you smoked cigarettes in your lifetime? In total, 15% out of 1566 participants reported having smoked cigarette at least once in their lifetime. Those with experience of social media were 2.52 times more likely (95% CI 1.72–3.69) to have experienced smoking cigarettes at least once compared to those who had no experience of smoking. In a multivariate nominal logistic regression model, three explanatory variables were statistically significant for lifetime experience of smoking cigarettes (*R*^2^ = 0.0767, *p* < 0.0001). Most significant was attending a private school; those attending private school were 3.21 more likely (95% CI 2.35–4.38; *p* < 0.0001) to have smoked compared to those attending a public school. Boys were also more likely to have smoked cigarettes compared to girls (OR 2.09, 95% CI 1.56–2.82; *p* < 0.0001). Finally, those with experience of social media were 1.91 times more likely (95% CI 1.29–2.83; *p* = 0.0013) to have smoked cigarettes compared to those who did not have that experience.

*Alcohol.* In the survey, the following question was asked: How often have you had a drink of alcohol of any kind in your lifetime? In total, 31% out of 1559 participants had the experience of drinking alcohol at least once in their lifetime. Participants with experience of social media were 1.47 times more likely (95% CI 1.15–1.90) to have drunk alcohol compared to those who reported no use of alcohol. In a multivariate nominal logistic regression model, four significant explanatory variables were identified for lifetime experience of drinking alcohol (*R*^2^ = 0.0405, *p* < 0.0001). These explanatory variables were attending a private school (OR 1.57, 95% CI 1.19–2.06; *p* = 0.0015), having at least one parent who had initiated or completed university or technical training (OR 1.57, 95% CI 1.17–2.12; *p* = 0.0026), not coming from a two-headed household (OR 1.51, 95% CI 1.18–1.96), and being born in Bissau (OR 1.48, 95 CI 1.10–2.00; *p* = 0.0102). There was a tendency for experience of social media to be associated with a lifetime experience of drinking alcohol, but it was not statistically significant (OR 1.29, 95% CI 0.96–1.73; *p* = 0.0900).

*Language proficiency*. Participants reported on their grade in Portuguese and English or French. There was no statistical association with good grades and the use of social media (OR 0.92, 95% CI 0.58–1.47; *p* = 0.7298).

## 4. Discussion

Here, we report results of a survey on the access to and use of digital technology among adolescents attending schools in the capital of Guinea-Bissau, Bissau. The key findings were that access to digital technologies was limited, even in this relatively privileged context. Mobile phones with internet capability were more commonly accessed, with about four out of five having experience of one, compared to the use of a laptop and/or desktop computer by fewer than half of the participants. The most significant explanatory variables of having access to these devices were attendance of a private school, coming from two-headed households and having educated parents—factors likely to be associated with increased wealth. Regarding usages of digital technology, a third of the survey participants reported they had not used the internet for any of the tools included in the survey, while the most commonly used tool was social media to connect with friends (two out of three sometimes doing this). The internet being used for study was applicable to just over half, with boys from wealthy families being most likely to do this. Boys were also most likely to have experience of any use of social media and to use the internet for games/entertainment/news. Effects of social media usage included being more likely to have participated in bullying behaviour, express increased feelings of anxiety and depression, and an increased likelihood to have tried smoking cigarettes, in line with results in other settings.

### 4.1. Access and Usage

The principal aim of the study was to explore digital technology access and usage of school-attending adolescents living in the capital city of Guinea-Bissau. Access to the primary three devices (desktop and/or laptop, and mobile phone with internet) was generally low (Table 2 and Table S2). Fifty-nine per cent of the respondents had never used a laptop in the last year, and 69% of them had never used a desktop computer. These results are in-line with data from other SSA countries; data from 2018 indicate that although access was improving, less than one out of ten households in SSA had access to a household computer [62]. The results reflect the pervasive poverty in the region that makes ownership of one only a distant dream for most families. Besides, the population of adolescents taken for our sample is likely to have given a more generous view of the situation, given that all the survey respondents were attending schools in the capital city, a privileged social setting. If samples were taken from adolescents generally, around the country, including rural areas and including those not enrolled in school, a different, bleaker picture would have almost certainly been painted.

In our survey, 79% of respondents reported using a mobile phone with internet capability in the last 12 months, a much higher proportion compared to laptop/desktop usage (Table 2 and Table S2). This is in line with other research in the field, with one study showing that most millennials in South Africa accessed the internet through a combination of mobiles and personal computers (88%) compared to 12% who only used a computer to access the internet [33]; mobile phones are cheap, easy to use and widely accessible in the market in contrast to expensive desktops or laptops, in addition to their dependence on stable access to electricity. Elsewhere on the continent, 74% of Zambians had mobile phones, but only 7% had internet access in the homes, with ownership levels for boys aged 15–19 years being higher (42%) than girls of the same age (32%) [32]. In Mozambique, another former Portuguese colony in SSA, a similar proportion (80% of urban households) were found to have mobile phone access [63], and in Liberia, 81% of urban households had mobile phone access, compared to only 10% having computer access in the house [64]. One potential reason for the more widespread usage of mobile phones compared to desktops and laptops is that mobiles are not considered the individual devices they are perceived to be in Europe, or the USA Phone sharing is a common concept in SSA, which inflates the proportion of populations that have mobile phone access, compared to computers, which are less portable and harder to share [17]. This is backed up by a 2018 study in South Africa which claimed that 76% of respondents who did not own a mobile phone had access to one through sharing [19]. Another study stated that 17% of students in South Africa said they shared a phone, which explained why despite 23% of respondents not owning a handset, 96% still claimed to use one on a typical day [28]. The proportion of phone sharing among adolescents in Guinea-Bissau is not known and needs further research.

Overall, internet access has been rapidly increasing with more than half of the global population estimated to have had access in 2018 [62]. The data expose, however, a global digital divide; in 2016, three out of four people had internet access in high-income countries, compared to less than one out of five in those least developed [9]. In SSA, South Africa was the most connected country with 54% of the population having internet access, compared to 4% for Guinea-Bissau [9]; more recent data indicate a gradual increase to 7.5% of the total Bissau-Guinean population [65]. Our data, from an albeit more privileged group, showed a more positive representation, with around two-thirds of students surveyed reporting some usage of the internet for one of seven listed activities (Table 4 and Table S3). 

Globally, around 40% of youth are said to use the internet to learn about things for school or health-related information, while only 9% of global youth said they used it to read about politics and improving the community [9]. Comparatively, our study showed that 55% of those who answered claimed some internet usage for study and 64% sometimes used the internet to read the news over the last 12 months. These were some of the higher categories for usage, with only social media usage to contact friends and family being more frequent (62% and 67%, respectively). These levels are in line with data from Tanzania, which showed that 65% of respondents used the internet to communicate with friends, while only 22% used it to read the news [66]. Furthermore, 15% of Tanzanian respondents reported internet usage to watch videos, and only 10% to play games. This is significantly lower than we found in our data from Guinea-Bissau, which showed that 51% had used the internet for entertainment in the last 12 months and 41% had used the internet to play games (predominantly a male behaviour). Reasons for this may be the privileged status of the study group, as well as the difference in wording to the questions. In our study, the adolescents were asked if they had ever used it within the last 12 months, while in Tanzania, it was more generally worded. 

Research has indicated that when Africans go online, they predominantly spend time on social media platforms, with other activities becoming less important [12]. This raises questions over whether the internet is more of a useful tool for education or simply a distraction. For example, some claim that the increasing use of phones by students causes more disruption in the classroom than good, with increases in late-night calls, bullying and harassment [23]. Our data do not indicate better grades in languages (Portuguese and English/French) for those with access to digital technology compared with those without access. This lack of association might indicate deficient knowledge on how to properly use the technology to improve individual skills, compounded further by lack of support and guidance from teachers who themselves often lack access and skills to positively benefit from it. This situation is further aggravated in the current COVID-19 pandemic, as estimates indicate that only 6% of pre-primary to upper secondary students in SSA can potentially be reached through the internet by remote learning policies [67].

### 4.2. Determinants of Usage and Barriers

Prevailing economic gaps have been identified as hindering access to digital technology [9]. Globally, SSA is the poorest region in the world; in 2013, about two-thirds of the region lived in multidimensional poverty, judged by monetary welfare measures (income and expenditure) and indicators on access to education and infrastructure [68]. In such a setting, the cost of digital technologies is an important barrier to usage. Across Africa, consumers of mobile data pay the highest cost relative to monthly income [69]. In terms of cost for a low-consumption mobile-data-and-voice basket (70 voice minutes, 20 SMS text messages, and 500 Mb broadband), in 2018 the costs in Guinea-Bissau were among the highest in the world; it ranked 175 out of 182 countries with costs equivalent to 26.3% of GNI (Gross National Income) per capita or USD 16.5 (USD 37.8 purchasing power parity) [70]. To determine whether wealth was a factor for Bissau-Guinean adolescents using mobile phones, we used two proxy indicators for a perceived actual wealth of their parents. Those who reported that their family could seldom or almost never afford a car or attended public school were significantly less likely than those from families who could afford to buy a car or attended a private school to use digital media for studies, social media, or entertainment (Table 5). In a multivariate nominal logistic regression model, the only statistically significant factor for parental wealth and use of these digital technologies was attending a private school compared to a public one. Similarly, adolescents attending private schools or whose parents could afford to buy a car were significantly more likely to have used a laptop and/or desktop computer (Table 3), which was also statistically significant in a multivariate model. Thus, the results manifest one important aspect of existing inequalities in this young population group.

Access to digital technology is not only shaped by wealth but also by gender. Overall, research has shown that men are more likely to adopt new technologies than women. In 2016, 28% of men in SSA had access to the internet compared to 22% females [14]. However, a few studies have pointed at rising equality, namely in South Africa, where there are equal numbers of male and female Facebook users [19]; in terms of phone ownership, female users reached parity in 2013/2014 with around half of both boys and girls between the ages of 9–18 owning mobile phones [23]. Data from eight SSA countries from the period 2017–2019 indicated that adolescent girls (aged 15–18 years) lagged behind boys in all the countries in information and communication technology (ICT) skills [71]. For example, in Ghana, 7% of the girls possessed such skills compared to 16% of boys, a divide that has to be taken into account when implementing remote learning policies during the COVID-19 pandemic. Our data show that, generally, there is little gender disparity among our sample group, although again, this may be due to the privileged population in focus. Of the students surveyed, approximately half of both boys and girls (53% and 49%, respectively) had an experience of having used either a desktop computer or a laptop (Table 2 and Table 3). There were slightly more boys (85%) who had ever used a mobile phone with an internet connection, compared to girls (75%), and the difference was statistically significant. Specifically looking at which gender is more likely to use the internet for help with their studies, boys (60%) were significantly more likely than girls to report doing so (Table 4 and Table 5). Similarly, boys were significantly more likely than girls to use social media, and the internet for entertainment, to play games, or to follow the news (Table 5).

In many SSA countries, usage of the technology brings out other gendered blocks than simple access. For example, in Tanzania, it was found that girls were scared of disclosing their interest in the internet to guardians and teachers [66]. This was due to usage by adolescent girls being less socially accepted than boys because of concerns over negative influences and cultural restrictions on the behaviour of girls, compared to boys. Indeed, 71% of internet users in Tanzania were male. In Malawi, only 1–2% of girls under the age of 19 in rural areas owned phones compared to 5% of boys, who were allowed to carry them freely [23]. Girls being seen carrying phones in public could see themselves being labelled as prostitutes because of social discourse. Similar themes were also found in Ghana, even in rural areas, over fears of phone ownership leading to teenage pregnancy. Although our data from Guinea-Bissau suggests that the levels of gender discrimination are not so high, the gap may be more pronounced in a different sample population, and with more specific questions being asked over digital media usage.

It has been alluded to in one study that education of the user and in particular, English language proficiency (in South Africa) was a key determinant of whether the respondent used the internet [16]. The logic behind this is that most of the content on the internet is in the major world languages, and thus if you do not speak these languages, you would be excluded from a lot of internet uses. In South Africa, about 20% of respondents said they could not easily read or write in English, and of those, only 3% used the internet. In our survey in Guinea-Bissau, rather than English proficiency, we compared this metric using Portuguese. Portuguese is the 9th most spoken language in the world [72], and we would hypothesise that having Portuguese spoken in the home (assuming that to equate to fluency in the language) should positively influence internet usage (Table 4). In our data, those who reported Portuguese being spoken at home were significantly more likely to use social media and the internet for entertainment, games and/or following the news (Table 5). The observed impact disappeared, however, in the multivariate model, thus explained by other factors than just language proficiency. 

Education has been identified as a major determinant of internet usage in the USA, with those with higher education more likely to adopt the technology compared to those with less education [73]. In Zambia, internet usage among women and men generally increased with better education, with 79% of women and 89% of men with higher education using the internet in the last 12 months compared to just 1% of women and 6% of men without higher education [32]. Our sample included school-attending adolescents; therefore, we can instead look at whether education of their parents affects internet usage. Adolescents who reported usage of the internet were overall more likely to have at least one parent who had started or completed university or technical training compared with those whose parents had less education (Table 5). However, such an impact disappeared in the multivariate nominal logistic regression model. 

One determinant of internet usage in SSA is whether you live in an urban area or a rural area. Rural African regions are characterised by low employment, a weak subsistence economy and poor technical infrastructure, negatively impacting ICT adoption [19]. In Zambia, 14% of urban populations had internet access compared to 2% of rural populations [32], while 82% of urban Mozambique populations had mobile phone access compared to 54% of rural households [63]. Our sample was taken from an urban area, making this difficult to measure using our data. As a proxy, we can, however, use data from where the students were born—split into two categories of inside the capital Bissau (68%) or outside (32%). Although this is not a perfect metric, because those born elsewhere could still be from other urban areas in the country, it may indicate wealth/habit of families using technology. Those who were born in Bissau were significantly more likely to report the use of a desktop computer and/or laptop compared to those born elsewhere (Table 3), and also in using social media and the internet for entertainment, games and/or following the news (Table 5). This impact, however, disappeared in our multivariate nominal logistic regression model.

### 4.3. Consequences of Technology Usage

The evidence for the influence of marketing through traditional media on adolescents’ use of tobacco is well documented [3,74]. In contrast, the impact of advertising on the consumption of alcohol has been found to be minimal in South Africa [75]. With internet access comes greater exposure to advertising, through online advertisements by social media influencers, on video platforms such as Facebook, YouTube and Instagram, and at the sides of most web pages. Another way the internet can influence tobacco or alcohol consumption is through peer pressure, in that those who are friends with other people who drink and smoke and see them doing so through social media channels, for example, may be more influenced to do so themselves [76]. Using our data, we asked the question: Does social media usage to connect with friends, and other interactions on the internet, impact usage of alcohol or smoking cigarettes? The criteria for social media usage for this analysis included those who answered that they had used any social media at least sometimes in the last 12 months; the criteria for alcohol, or smoking cigarettes were lifetime experience in use. Adolescents who reported lifetime experience of either smoking cigarettes or consuming alcohol were significantly more likely to have used social media compared to those who did not have that experience; this association held up in a multivariate nominal logistic regression model for smoking cigarettes but not for lifetime experience of drinking alcohol. However, our bivariate results indicate support to another study that suggested that the rise of social media usage sees more young people exposed to and displaying pro-alcohol messages [77]. Social media sites, such as Facebook and Twitter, have conducive formats for sharing pictures with a culture of anonymity and privacy, and posted content is likely to be seen not only by peers but by younger users, who are more impressionable. Moreno and Whitehill [77] cited several studies that referred to alcohol-related displays on social media, including photographs depicting or alluding to alcohol consumption and links to alcohol-related groups or companies. Although this may remain relevant for smoking, as well as for alcohol abuse, what seems to have more of an impact are movie portrayals of smoking, as well as peer and parent smoking [78]. Social media usage is obviously linked with internet access, and with that comes more exposure to both peer activities and also media (music videos and movies). Fifty-one percent of survey participants in Guinea-Bissau had an experience of using the internet for entertainment, which includes these activities (Table 4). 

In addition to alcohol and smoking, some sources claim that digital technology adoption intensifies cases of bullying in children, which is known to affect wellbeing [9]. Adolescents in Bissau who had experience of using social media were more than twice as likely to have participated in a group that teased others. Social media and other online platforms provide easier access to others, and over time bullying can become normalised and even rewarded with increased social status [79]. Furthermore, the veil of anonymity that the internet and social media can provide can also lead to more bullying, because the lack of face-to-face encounters prevents many of the negative consequences of bullying from occurring. Another study from South Africa showed that one in three children had experienced cyberbullying and that 47% of those had been bullied through a mobile phone, showing that the devices are being used as a tool to increase bullying frequency [10]. In our data, social media usage was statistically the most important factor linked to bullying, with other strong explanatory factors being attending a private school and having educated parents. These two factors, as well as having internet access, are associated with wealth, therefore there is a case for saying that it is the relative wealth of the adolescents, strengthened by the actual usage of social media, which is causing the increased participation in bullying behaviour. 

Bullying is most common in the west and central African regions, with five of the top ten countries for bullying rates (65–81%) being situated in Africa [80]. Girls reported more indirect bullying, while boys experienced direct bullying. Our data from Bissau showed little gender differences. Boys were marginally more likely to have participated in bullying behaviour than girls, although we lack data on the gender of the victims and the exact nature of the bullying, themes that merit further research. Overall, only 10% of boys and 11% of girls had participated in the teasing of another individual. These bullying rates are relatively low compared to the rest of Africa, which could be due to the fact that the line of the questioning came from an accusatory angle, rather than an experience angle—i.e., if the question had been phrased “How often have you been teased by another group?”, the percentages might be higher. 

In terms of mental health, increased usage of digital technologies, in particular, the usage of social media, has been said to also increase anxiety and low self-esteem [81,82]. According to one study, one potential hazard of social media is a growing preoccupation with body image, through exposure to media output on glamorous celebrities and comparison with friends on social media [7]. It has also been reported that excessive use of digital technology can contribute to childhood depression and anxiety, due to the immersion in screens creating an internet dependency [9]. Although 8% of the participants in Guinea-Bissau had said they often felt tense in the past week, they were almost twice as likely to report tension if they had experience of using social media—interestingly, the only statistically significant explanatory variable. This conclusion supports the data from UNICEF and shows that social media might impact negatively on mental wellbeing [9]. Similarly, with depression, adolescents in our survey who used social media were significantly more likely to have felt sad or blue in the last week, compared to those that had not. This result again is supported by other literature [83]. Facebook and Instagram usage has been directly and indirectly correlated with symptoms of depression, although the seriousness of symptoms increases with age [84]. These findings beg the question: are adolescents in SSA at a higher risk of suffering from mental health conditions due to newly increased usage of these tools?

Our results highlight the need for increased awareness among Bissau-Guinean adolescents on the potentially negative consequences of the usage of digital media. Not all are familiar with the technology, a fact that is likely to apply as well to parents and teachers alike. For preventive measures, the most effective platforms include TV or radio programmes, for example, supported with data from the survey. Parents also need to be made aware of the potential negative consequences of the usage of the technology in the daily lives of their children [85], and by practising kind parenting styles, they could also become more effective in preventing cyberbullying against or by their children [86]. Furthermore, school headmasters and teachers could engage in kind and reasoning discussion with students in class as a preventive measure against cyberbullying, to improve their health and wellbeing in the digital age. Interestingly, SSA has been identified as the next big market by international operators of gambling [27], an additional threat to Bissau-Guinean adolescents of which parents and teachers should be made aware of and needs further research in the setting. Finally, concerted actions are also needed by social media companies, such as Facebook and Instagram, to develop internet policies in collaborative partnerships that protect children from cyberspace risks [87].

### 4.4. Strength and Limitations

One strength of the present study builds on its reaching out to school-attending adolescents in the capital Bissau, randomly selected for participation from a specially compiled register targeting adolescents aged 15–16 years, in both public and private schools. The survey methodology built on gained experience within the Planet Youth collaboration with similar surveys having been implemented in 32 countries and 111 communities [88]. It is the first study of its kind in the setting. However, there are limitations. Ethnicity and religion were not included in the statistical analysis because the application is complex in the context of multi-ethnic Guinea-Bissau, and results do not render themselves for a proper interpretation. Another limitation is that the study included only school-attending adolescents, a privileged urban group in the setting, thus excluding out-of-school adolescents, and those living outside the capital Bissau. 

## 5. Conclusions

The main conclusion of our study is that even in this privileged group of adolescents in the capital Bissau, access to digital technology is low in Guinea-Bissau. This provides more evidence to the existing global digital divide, in addition to the gender gap in the usage of the technology. The evidence seems to link predominantly negative consequences to the usage of the devices, in particular social media. Still, these are problems adolescents also face in Europe and other high-income countries and are an expression of commonality of adolescents’ experiences across different world regions. This does not equate to digital technology being bad. The lesson to be learned is, therefore, about educating the users of the technology to obtain the biggest benefits. Adolescents who learn to use the internet to search for information, help with studies, and gain experience using tools such as word processors will be better equipped for future studies and employment. An inevitable consequence of increased media usage is that adolescents will face higher exposure to advertisements, potentially for harmful products such as alcohol and tobacco, and peers and celebrities engaging in activities we do not wish to see adolescents’ replicate. However, if used correctly, the benefits of the internet and the technologies to increase communication has the potential to create more benefits than negatives. Since the adoption of the SDGs in 2015, the needs of adolescents—the SDG generation—have increasingly been in focus. Our results indicate that Bissau-Guinean adolescents are at a large risk of being left behind without improved opportunities to develop their digital skills and proficiency in preparation for a labour market that increasingly demands such experience and knowledge.

## Figures and Tables

**Table 1:** Table with selected socioeconomic variables of participants’, by gender *. Adolescents attending schools in Bissau, June 2017.

Characteristics	Total *n* (%)	Boy *n* (%)	Girl *n* (%)
Are you a boy or a girl?	1978 (100)	954 (48)	1024 (52)
Age (years)			
14	130 (7)	55 (6)	75 (8)
15	346 (19)	166 (19)	180 (19)
16	435 (24)	194 (22)	241 (26)
17	581 (32)	305 (35)	276 (29)
18	288 (16)	139 (16)	149 (16)
19+	45 (2)	22 (2)	23 (2)
Total	1780 (100)	859 (100)	921 (100)
If you were born in Guinea-Bissau, choose the region where you were born			
Bissau	1262 (69)	609 (68)	653 (69)
Outside Bissau	580(31)	293 (32)	287 (31)
Total	1842 (100)	902 (100)	940 (100)
Type of school			
Public	1033 (52)	530 (56)	503 (49)
Private	945 (48)	424 (44)	521 (51)
Total	1978 (100)	954 (100)	1024 (100)
Household characteristics *			
Two-headed family	955 (55)	491(28)	464 (27)
At least one parent who has started or	1091 (61)	508 (29)	583 (33)
completed university or technical training			
At least one parent who works	1595 (85)	759 (40)	836 (45)
Portuguese spoken at home	108 (6)	44 (3)	64 (4)
Family financial situation *			
Better compared to peers	1600 (85)	754 (40)	846 (45)
Cannot afford a car	760 (48)	384 (24)	376 (24)

* See Table S1 for information on selected survey questions with response alternatives and frequency of responses on the socioeconomic situation of the participants, by gender.

**Table 2:** Number and percentage of respondents who responded to the following question: “What kind of information technology do you generally use?”, by gender. Adolescents attending schools in Bissau, June 2017.

Frequency of Using the Technology	Total *n* (%)	Boys *n* (%)	Girls *n* (%)
Desktop computer			
Every day	109 (9)	51 (8)	58 (9)
2–3 times/week	110 (9)	62 (10)	48 (8)
Every week	105 (8)	50 (8)	55 (9)
Less than monthly	70 (6)	33 (5)	37 (6)
Never	873 (69)	430 (69)	443 (69)
Total	1267 (100)	626 (100)	641 (100)
Laptop computer			
Every day	194 (14)	96 (14)	98 (14)
2–3 times/week	155 (11)	84 (12)	71 (10)
Every week	145 (10)	72 (12)	73 (10)
Less than monthly	86 (6)	43 (6)	43 (6)
Never	824 (59)	392 (57)	432 (60)
Total	1404 (100)	687 (100)	717 (100)
Mobile phone with internet			
Every day	566 (34)	286 (35)	280 (34)
2–3 times/week	364 (22)	193 (24)	171 (21)
Every week	308 (19)	170 (21)	138 (17)
Less than monthly	72 (4)	36 (4)	36 (4)
Never	343 (21)	138 (17)	205 (25)
Total	1653 (100)	823 (100)	830 (100)

**Table 3:** Bivariate analysis for explanatory socioeconomic variables for experience in the use of computers and/or laptops, and mobile phones with internet capabilities. Adolescents attending schools in Bissau, June 2017 *.

Socioeconomic Variables	Desktop Computer and/or Laptop		Mobile Phone with Internet Capabilities	
	*p*-Value	OR (95% CI)	*p*-Value	OR (95% CI)
Are you a boy or a girl?				
Girl	0.2431	0.89 (0.72–1.09)	<0.0001	0.61 (0.48–0.78)
Boy		1		1
In what region were you born?				
Bissau	0.0069	1.36 (1.09–1.71)	0.1723	1.19 (0.93–1.54)
Elsewhere		1		1
Attendance by school type				
Private school	<0.0001	2.55 (2.07–3.13)	<0.0001	1.87 (1.46–2.38)
Public school		1		1
Household				
Two-headed family	<0.0001	1.60 (1.29–1.99)	0.3596	1.12 (0.87–1.45)
Other composition		1		1
Employment status of parents				
At least one parent works	0.0067	1.50 (1.12–2.02)	0.0876	1.33 (0.96–1.84)
Other arrangements		1		1
Education of parents				
At least one parent who has started or completed university education or technical training	<0.0001	2.11 (1.69–2.63)	0.0104	1.39 (1.08–1.78)
Other or no education		1		1
Languages spoken at home				
Portuguese	<0.0001	2.97 (1.80–4.90)	0.0322	1.98 (1.01–3.88)
Other languages		1		1
How well financially do you think your family is in comparison to other families in your neighbourhood?				
A little better, considerably better, or much better off compared to peers	0.0152	1.43 (1.07–1.91)	0.7139	0.94 (0.66–1.33)
Similar to others, or a little, considerably or much worse off		1		1
My parents can afford to buy a car				
Seldom or almost never	<0.0001	0.43 (0.35–0.54)	0.0015	0.66 (0.52–0.86)
Sometimes, often, or almost always		1		1

* See Table S1 for survey questions used in the analysis.

**Table 4:** Participants’ frequency of usage of different tools of digital technology, by gender. Adolescents attending schools in Bissau, June 2017.

Frequency of Using Technology	Total *n* (%)	Boys *n* (%)	Girls *n* (%)
Internet for studies			
Every day	371 (22)	197 (24)	166 (20)
2–3 times/week	284 (17)	151 (18)	127 (15)
Every week	183 (11)	99 (12)	81 (10)
Less than monthly	95 (6)	47 (6)	44 (5)
Never	772 (45)	332 (40)	422 (50)
Total	1705 (100)	826 (100)	840 (100)
Internet for entertainment			
Every day	228 (16)	118 (17)	108 (16)
2–3 times/week	216 (16)	131 (19)	85 (13)
Every week	166 (12)	90 (13)	70 (10)
Less than monthly	102 (7)	54 (8)	44 (7)
Never	675 (49)	297 (43)	365 (54)
Total	1387 (100)	690 (100)	672 (100)
Social media to connect with friends			
Every day	269 (18)	138 (19)	125 (17)
2–3 times/week	275 (19)	155 (21)	112 (16)
Every week	278 (19)	148 (20)	125 (17)
Less than monthly	94 (6)	51 (7)	41 (6)
Never	570 (38)	242 (33)	316 (44)
Total	1486 (100)	734 (100)	719 (100)
Using social media to connect with the family			
Every day	283 (18)	138 (18)	139 (19)
2–3 times/week	311 (20)	164 (22)	143 (19)
Every week	331 (22)	177 (24)	147 (20)
Less than monthly	104 (7)	50 (7)	49 (7)
Never	510 (33)	225 (30)	275 (37)
Total	1539 (100)	754 (100)	753 (100)
Using social media to connect with people you would like to know			
Every day	211 (15)	115 (16)	93 (13)
2–3 times/week	231 (16)	134 (19)	93 (13)
Every week	187 (13)	106 (15)	76 (11)
			
Less than monthly	140 (10)	81 (11)	56 (8)
Never	672 (47)	284 (39)	375 (54)
Total	1441 (100)	720 (100)	693 (100)
Using internet to play games			
Every day	194 (14)	113 (16)	74 (11)
2–3 times/week	154 (11)	102 (14)	50 (7)
Every week	146 (10)	99 (14)	42 (6)
Less than monthly	94 (7)	51 (7)	41 (6)
Never	854 (59)	359 (50)	481 (70)
Total	1442 (100)	724 (100)	688 (100)
Using internet to follow news			
Every day	365 (24)	201 (26)	152 (20)
2–3 times/week	254 (16)	137 (18)	112 (15)
Every week	268 (17)	152 (20)	114 (15)
Less than monthly	115 (7)	63 (8)	47 (6)
Never	551 (36)	210 (28)	329 (44)
Total	1553 (100)	763 (100)	754 (100)

**Table 5:** Usage of digital technology by selected explanatory socioeconomic variables. Adolescents in schools in Bissau, June 2017 *.

Socioeconomic Variables *	Studies		Social Media		Entertainment, News and/or Games	
	*p*-Value	OR (95% CI)	*p*-Value	OR (95% CI)	*p*-Value	OR (95% CI)
Are you a boy or a girl?						
Girl	<0.0001	0.67 (0.55–0.81)	0.0012	0.69 (0.56–0.87)	<0.001	0.43 (0.34–0.55)
Boy		1		1		1
In what region were you born?						
Born in Bissau	0.9317	1.01 (0.82–1.25)	0.0006	1.51 (1.19–1.91)	0.0007	1.52 (1.20–1.94)
Born elsewhere		1		1		1
Attendance by school type						
Private school	0.0313	1.23 (1.02–1.49)	<0.0001	1.93 (1.55–2.42)	<0.0001	1.77 (1.40–2.23)
Public school		1		1		1
Household composition						
Two-headed family	0.0190	1.28 (1.04–1.56)	0.3990	1.10 (0.88–1.39)	0.1247	1.21 (0.95–1.54)
Other composition		1		1		1
Employment status of parents						
At least one parent works	0.1381	1.23 (0.94–1.62)	0.0291	1.41 (1.04–1.91)	0.0760	1.33 (0.97–1.83)
Other arrangements		1		1		1
Education of parents						
At least one parent who has started or completed university education or technical training	0.0917	1.19 (0.97–1.47)	0.0038	1.41 (1.12–1.78)	0.0012	1.49 (1.17–1.90)
Other education		1		1		1
Languages spoken at home						
Portuguese	0.1449	1.39 (0.89–2.16)	0.0261	1.81 (1.04–3.16)	0.0358	1.88 (1.00–3.53)
Other languages		1		1		1
How well financially do you think your family is in comparison to other families in your neighbourhood?						
A little better, considerably better, or much better off compared to peers	0.7593	0.96 (0.73–1.26)	0.9731	1.01 (0.74–1.37)	0.5712	1.10 (0.80–1.51)
Similar to others, or a little, considerably or much worse off		1		1		1
My parents can afford to buy a car						
Seldom or almost never	0.0099	0.76 (0.62–0.94)	<0.0001	0.60 (0.48–0.77)	0.0112	0.73 (0.57–0.93)
Sometimes/often/almost always		1		1		1

* See Table S1 for the wording of the survey questions.

## Supplementary Materials

The following are available online at https://www.mdpi.com/1660-4601/17/23/8937/s1, Table S1: Selected survey questions on socioeconomic background of respondents. Adolescents attending schools in Bissau, June 2017. Table S2: Number and percentage of respondents who responded to the following question: “What kind of information technology do you generally use?”, by public and private school. Adolescents attending schools in Bissau, June 2017. Table S3: Participants’ frequency of usage of different tools of digital technology, by public or private school. Adolescents attending schools in Bissau, June 2017.
