# Supplementary material for: Use of Digital Technology among Adolescents Attending Schools in Bissau, Guinea-Bissau
Source: Int J Environ Res Public Health. 2020 Dec 1;17(23):8937. doi: 10.3390/ijerph17238937 (PMC7729758; doi:10.3390/ijerph17238937)
Supplement: Supplementary file 1 [file ijerph-17-08937-s001.pdf]

Table S1. Selected survey questions on socioeconomic background of respondents. Adolescents attending schools in Bissau, June 2017.

Questions and Response Alternatives	Total *n* (%)	Boy *n* (%)	Girl *n* (%)
**Which of the following persons live in your home?**			
Both parents	859 (49)	445 (52)	414 (47)
Mother, but not with father	449 (26)	198 (23)	251 (28)
Father, but not with mother	138 (8)	70 (8)	68 (8)
Mother and with her partner	51 (3)	22 (3)	29 (3)
Father and his new partner	45 (3)	24 (3)	21 (2)
I live with friends	8 (0)	7 (1)	1 (0)
I live with my grandparents	161 (9)	77 (9)	84 (9)
I live in different arrangements	36 (2)	14 (2)	22 (2)
Total	1747 (100)	857 (100)	890 (100)
**What is the highest level of schooling your mother completed?**			
Graduated from a college/university	317 (17)	249 (28)	68 (7)
Started college/university but has not finished	128 (7)	74 (8)	54 (6)
Graduated from technical training school	144 (8)	113 (13)	31 (3)
Started technical training school but has not finished	52 (3)	28 (3)	24 (3)
Secondary education (10–12 grade)	281 (15)	122 (14)	159 (17)
Primary education (7–9 grade)	297 (16)	91 (10)	206 (22)
Primary education (5–6 grade)	111 (6)	31 (3)	80 (9)
Primary education (1–4 grade)	128 (7)	36 (4)	92 (10)
Does not read or write	170 (9)	48 (5)	122 (13)
Other	6 (0)	4 (0)	2 (0)
I don´t know	188 (10)	95 (11)	93 (10)
Total	1822 (100)	891 (100)	931 (100)
**What is the highest level of schooling your father completed?**			
Graduated from a college/university	483 (27)	215 (25)	268 (30)
Started college/university but has not finished	70 (4)	28 (3)	42 (5)
Graduated from junior college or trade school	422 (24)	204 (24)	218 (24)
Started technical training school but has not finished	52 (3)	26 (3)	26 (3)
Secondary education (10–12 grade)	267 (15)	137 (16)	130 (14)
Primary education (7–9 grade)	151 (9)	78 (9)	73 (8)
Primary education (5–6 grade)	54 (3)	27 (3)	27 (3)
Primary education (1–4 grade)	80 (5)	41 (5)	39 (4)
Does not read or write	56 (3)	31 (4)	25 (3)
Other	11 (1)	5 (1)	6 (1)
I don´t know	111 (6)	61 (7)	50 (6)
Total	1757 (100)	853 (96)	904 (100)
**Does your mother work outside the home?**			
Mother works at home with domestic tasks	700 (38	363 (41)	337 (36)
Mother works part-time outside the home	452 (25)	217 (24)	235 (25)
Mother works full-time outside the home	411 (22)	183 (21)	228 (24)
Mother is unemployed	76 (4)	41 (5)	35 (4)
Mother is disabled	7 (0)	2 (0)	5 (1)
Mother has retired	16 (1)	9 (1)	7 (1)
Mother is studying	80 (4)	24 (3)	56 (6)
Mother is studying and working outside the home	57 (3)	27 (3)	30 (3)
I don´t know	38 (2)	23 (3)	15 (2)
Total	1837 (100)	889 (100)	948 (100)
**Does your father work outside the home?**			
Father works at home with domestic tasks	134 (7)	64 (7)	70 (8)
Father works part-time outside the home	286 (16)	146 (17)	140 (15)
Father works full-time outside the home	1056 (58)	498 (57)	558 (60)
Father is unemployed	69 (4)	37 (4)	32 (3)
Father is disabled	10 (1)	8 (1)	2 (0)
Father has retired	62 (3)	32 (4)	30 (3)
Father is studying	33 (2)	15 (2)	18 (2)
Father is studying and working outside the home	84 (5)	39 (4)	45 (5)
I don´t know	74 (4)	37 (4)	37 (4)
Total	1808 (100)	876 (100)	932 (100)
**What languages are spoken in your home?**			

Balanta	77 (5)	38 (5)	39 (5)
Beafada	22 (1)	11 (1)	11 (1)
Bijagós	6 (0)	4 (0)	2 (0)
Crioulo	1101 (66)	537 (65)	564 (66)
Felupe	17 (1)	8 (1)	9 (1)
Fula	105 (6)	61 (7)	44 (5)
Mancanha	50 (3)	24 (3)	26 (3)
Mandinga	82 (5)	40 (5)	42 (5)
Manjaco	67 (4)	36 (4)	31 (4)
Nalu	2 (0)	1 (0)	1 (0)
Papel	31 (2)	13 (2)	18 (2)
Portuguese	108 (6)	44 (5)	64 (7)
Sussu	3 (0)	1 (0)	2 (0)
Other	9 (1)	4 (0)	5 (1)
Total	1680 (100)	822 (100)	858 (100)
**How well-off financially do you think your family is in comparison to other families in your neighbourhood?**			
Much better off	228 (25)	273 (28	501 (27)
Considerably better off	100 (11)	161 (17)	261 (14)
A little better off	426 (47)	412 (43)	838 (45)
Similar to others	65 (7)	52 (5)	117 (6)
A little worse off	61 (7)	51 (5)	112 (6)
Considerably worse off	6 (1)	4 (0)	10 (1)
Much worse off	18 (2)	15 (2)	33 (2)
Total	904 (100)	968 (100)	1872 (100)
**Please state if and to what extent the following applies to you: My parents can afford to buy a car.**			
Almost never	622 (40)	325 (42)	297 (37)
Seldom	138 (9)	59 (8)	79 (10)
Sometimes	382 (24)	192 (25)	190 (24)
Often	239 (15)	124 (16)	115 (14)
Almost always	191 (12)	77 (10)	114 (14)
Total	1572 (100)	777 (100)	795 (100)

Table 2. Number and percentage of respondents who responded to the following question: "What kind of information technology do you generally use?", by public and private school. Adolescents attending schools in Bissau, June 2017.

Frequency of Using Technology	Total *n* (%)	Public School *n* (%)	Private School *n* (%)
**Desktop computer**			
Every day	111 (9)	65 (9)	46 (8)
2–3 times/week	113 (9)	60 (8)	53 (9)
Every week	107 (8)	45 (6)	62 (11)
Less than monthly	70 (5)	20 (3)	50 (9)
Never	892 (69)	522 (73)	370 (64)
Total	1293 (100)	712 (100)	581 (100)
**Laptop computer**			
Every day	200 (14)	89 (12)	111 (16)
2–3 times/week	163 (11)	72 (9)	91 (14)
Every week	146 (10)	51 (7)	95 (14)
Less than monthly	86 (6)	25 (3)	61 (9)
Never	844 (59)	528 (69)	316 (47)
Total	1439 (100)	765 (100)	674 (100)
**Mobile phone with internet**			
Every day	584 (34)	323 (36)	261 (33)
2–3 times/week	374 (22)	171 (19)	203 (25)
Every week	317 (19)	147 (16)	170 (21)
Less than monthly	72 (4)	30 (3)	42 (5)
Never	351 (21)	228 (25)	123 (15)
Total	1698 (100)	899 (100)	799 (100)

Table 3. Participants' frequency of usage of different tools of digital technology, by public or private school. Adolescents attending schools in Bissau, June 2017.

Frequency of Using Technology	Total *n* (%)	Public School *n* (%)	Private School *n* (%)
**Internet for studies**			
Every day	371 (22)	233 (26)	138 (17)
2–3 times/week	284 (17)	125 (14)	159 (20)
Every week	183 (11)	85 (9)	98 (12)
Less than monthly	95 (6)	34 (4)	61 (8)
Never	772 (45)	435 (48)	337 (42)
Total	1705 (100)	912 (100)	793 (100)
**Internet for entertainment**			
Every day	228 (16)	108 (15)	120 (18)
2–3 times/week	216 (16)	99 (14)	117 (18)
Every week	166 (12)	72 (10)	94 (14)
Less than monthly	102 (7)	39 (5)	63 (10)
Never	675 (49)	414 (57)	261 (40)
Total	1387 (100)	732 (100)	655 (100)
**Social media to connect with friends**			
Every day	269 (18)	138 (18)	131 (19)
2–3 times/week	275 (19)	124 (16)	151 (21)
Every week	278 (19)	133 (17)	145 (21)
Less than monthly	94 (6)	37 (5)	57 (8)
Never	570 (38)	348 (45)	222 (31)
Total	1486 (100)	780 (100)	706 (100)
**Using social media to connect with family**			
Every day	283 (18)	144 (18)	139 (19)
2–3 times/week	311 (20)	151 (19)	160 (22)
Every week	331 (22)	156 (19)	175 (24)
Less than monthly	104 (7)	37 (5)	67 (9)
Never	510 (33)	321 (40)	189 (26)
Total	1539 (100)	809 (100)	730 (100)
**Using social media to connect with people you'd like to know**			
Every day	211 (15)	134 (17)	77 (12)
2–3 times/week	231 (16)	119 (15)	112 (17)
Every week	187 (13)	99 (13)	88 (13)
Less than monthly	140 (10)	55 (7)	85 (13)
Never	672 (47)	365 (47)	307 (46)
Total	1441 (100)	772 (100)	669 (100)
**Using the internet to play games**			
Every day	194 (13)	215 (26)	87 (13)
2–3 times/week	154 (11)	118 (14)	75 (11)
Every week	146 (10)	141 (17)	78 (12)
Less than monthly	94 (7)	50 (6)	65 (10)
Never	854 (59)	297 (36)	368 (55)
Total	1442 (100)	821 (100)	673 (100)
**Using the internet to follow the news**			
Every day	365 (24)	215 (26)	150 (20)
2–3 times/week	254 (16)	118 (14)	136 (19)
Every week	268 (17)	141 (17)	127 (17)
Less than monthly	115 (7)	50 (6)	65 (9)
Never	551 (35)	297 (36)	254 (35)
Total	1553 (100)	821 (100)	732 (100)
